# Radiotherapy Strategies for WHO Grade 1 Meningioma: A Systematic Review and Meta-Analysis Comparing Proton Therapy, Conventional Radiotherapy, and Radiosurgery

**DOI:** 10.3390/cancers18152506

**Published:** 2026-08-05

**Authors:** Hazuki Nitta, Masashi Mizumoto, Kazushi Maruo, Yoshiko Oshiro, Yinuo Li, Kenji Kagawa, Takashi Saito, Haruko Numajiri, Kei Nakai, Tetsuo Nonaka, Hideyuki Sakurai

**Affiliations:** 1 Department of Radiation Oncology, University of Tsukuba, Tsukuba 305-8576, Ibaraki, Japan; hnitta@pmrc.tsukuba.ac.jp (H.N.); ooyoshiko@pmrc.tsukuba.ac.jp (Y.O.); yli@pmrc.tsukuba.ac.jp (Y.L.); saitoh@pmrc.tsukuba.ac.jp (T.S.); haruko@pmrc.tsukuba.ac.jp (H.N.); knakai@pmrc.tsukuba.ac.jp (K.N.); hsakurai@pmrc.tsukuba.ac.jp (H.S.); 2 Department of Radiation Oncology, Japanese Red Cross Medical Center, Shibuya, Tokyo 150-8935, Japan; nonaka@pmrc.tsukuba.ac.jp; 3 Department of Biostatistics, Institute of Medicine, University of Tsukuba, Tsukuba 305-8576, Ibaraki, Japan; maruo@md.tsukuba.ac.jp; 4 Department of Radiation Oncology, Tsukuba Medical Center Hospital, Tsukuba 305-8558, Ibaraki, Japan; 5 Department of Neurosurgery, Japanese Red Cross Medical Center, Shibuya, Tokyo 150-8935, Japan; kagawa_kenji@med.jrc.or.jp

**Keywords:** meta-analysis, proton therapy, photon radiotherapy, meningioma, outcomes

## Abstract

Background/Objectives: The optimal radiotherapy modality for World Health Organization (WHO) Grade 1 meningioma remains uncertain. Proton therapy offers dosimetric advantages through reduced radiation exposure to normal brain tissue, but comparative clinical data across contemporary radiotherapy modalities are limited. We performed a systematic review and meta-analysis comparing local control (LC) outcomes among conventionally fractionated proton therapy (CF-PT), conventionally fractionated photon radiotherapy (CF-Photon RT), Linac-based stereotactic radiosurgery/radiotherapy (Linac-based SRS/SRT), and Gamma Knife radiosurgery (GKRS). Methods: A systematic literature search of PubMed (2000–2024) was conducted to identify studies reporting LC for WHO Grade 1 meningioma treated with CF-PT, CF-Photon RT, Linac-based SRS/SRT, or GKRS. Random-effects meta-analyses were performed to estimate pooled 1- to 5-year LC rates. Random-effects meta-regression analyses were conducted using modality, age, sex, and gross tumor volume (GTV) as covariates. Results: Twenty-four studies comprising 4673 patients were included in the meta-analysis. Median GTVs were larger in the CF-PT and CF-Photon RT cohorts than in the Linac-based SRS/SRT and GKRS cohorts. All modalities achieved excellent LC: 5-year LC rates were 95.9% for CF-PT, 93.0% for CF-Photon RT, 95.3% for Linac-based SRS/SRT, and 89.8% for GKRS. Meta-regression showed no significant association between modality or GTV and LC. In the CF-PT cohort, 5-year overall survival was 93.1%. Conclusions: Major radiotherapy modalities achieved excellent and comparable LC. Notably, CF-PT and CF-Photon RT maintained favorable LC despite being used for larger tumors than stereotactic approaches. Future studies are needed to evaluate long-term tumor control and treatment-related toxicity to define optimal treatment strategies.

## Simple Summary

This study is the first meta-analysis comparing local control (LC) outcomes among conventionally fractionated proton therapy (CF-PT), conventionally fractionated photon radiotherapy, and stereotactic approaches for WHO Grade 1 meningioma. Excellent LC was achieved across all modalities, with no significant differences despite variations in tumor volume. Notably, CF-PT achieved a 5-year LC rate of 95.9%, comparable to contemporary photon-based approaches while being applied to larger tumors. Given the prolonged survival expected for most patients with benign meningiomas, treatment selection should consider both tumor control and long-term survivorship, including preservation of quality of life and reduction in late toxicities. Future studies are needed to evaluate treatment-related toxicity and long-term clinical outcomes to refine optimal treatment strategies.

## 1. Introduction

Meningiomas originate from arachnoid cap cells of the arachnoid membrane and are typically dural-based tumors. They are the most common primary brain tumor and predominantly affect middle-aged and older adults, with a higher incidence in females [1]. These tumors may arise at any site where dura mater is present, most commonly along the cerebral convexity, parasagittal region, and falx cerebri [2]. Clinical manifestations include focal neurological deficits, symptoms of increased intracranial pressure, and epilepsy, although an increasing proportion of lesions are now detected incidentally due to advances in neuroimaging [3,4]. Histopathologically, meningiomas are classified into World Health Organization (WHO) Grades 1–3, which are associated with recurrence risk and guide therapeutic decision making [5]. Most meningiomas are WHO Grade 1 tumors and have favorable long-term survival, whereas higher-grade lesions show more aggressive behavior and a greater risk of recurrence. The etiology remains incompletely understood, but hormonal influences have been implicated, and meningiomas may also arise as secondary neoplasms following prior cranial irradiation [6,7,8].

Management strategies for meningiomas are individualized according to patient age, comorbidities, symptoms, tumor size, location, histological grade, and expected long-term outcomes. Observation is generally recommended for small asymptomatic lesions [9]. However, active treatment should be considered for younger patients and for tumors with radiographic features associated with progression, including larger size, absence of calcification, T2 hyperintensity, or peritumoral edema [4,10]. Surgical resection remains the cornerstone of treatment whenever feasible. Radiotherapy plays an important role for surgically challenging tumors, residual disease after subtotal resection, recurrent lesions, and selected high-grade meningiomas [9]. Radiotherapy approaches include conventionally fractionated radiotherapy, stereotactic radiosurgery, and stereotactic radiotherapy, delivered using photon- or proton-based technologies. Despite widespread clinical use, there is no clear consensus on the optimal radiotherapy modality or fractionation strategy for WHO Grade 1 meningioma.

Because patients with WHO Grade 1 meningioma frequently have long-term survival extending over several decades, treatment selection should consider both tumor control and the potential risk of late treatment-related toxicities. Radiation-induced neurocognitive decline, cerebrovascular complications, and secondary malignancies have become increasingly important concerns in long-term survivors. Proton therapy (PT) offers a dosimetric advantage through reduction in unnecessary radiation exposure to surrounding normal brain tissue. This advantage may be particularly relevant for large or anatomically complex meningiomas requiring irradiation near critical organs at risk. However, evidence supporting the clinical role of PT in meningioma is limited, and comparative data across contemporary radiotherapy modalities are scarce. Furthermore, it remains unclear whether differences in treatment modality translate into clinically meaningful differences in tumor control for WHO Grade 1 meningioma.

With this background, we performed a systematic review and meta-analysis comparing conventionally fractionated proton therapy (CF-PT), conventionally fractionated photon radiotherapy (CF-Photon RT), Linac-based stereotactic radiosurgery/radiotherapy (Linac-based SRS/SRT), and Gamma Knife radiosurgery (GKRS) for WHO Grade 1 meningioma. The primary objective was to evaluate local control (LC) across modalities and to define the potential role of PT.

## 2. Materials and Methods

### 2.1. Selection Criteria for Meta-Analysis

This study was conducted in accordance with the Preferred Reporting Items for Systematic Reviews and Meta-Analyses (PRISMA) guidelines and recommendations [11]. The review was not registered, and no formal protocol was prepared. Only articles written in English were included. Article screening was performed independently by two reviewers (HN and MM), and discrepancies were resolved through discussion. YL assisted with the literature search and screening process. One reviewer (HN) extracted data following a standardized data extraction form that included the author, year of publication, follow-up period, treatment details, patient characteristics, and outcomes. A second reviewer (MM) verified the accuracy of the extracted data. Inclusion criteria were: (1) clinical or pathological diagnosis of WHO Grade 1 meningioma; (2) treatment with radiotherapy, including CF-PT, CF-Photon RT, Linac-based SRS/SRT, or GKRS; and (3) availability of LC data following radiotherapy. WHO Grades 2 and 3 meningiomas were not included because the number of studies for each radiotherapy modality was limited and heterogeneity in patient background and treatment indications precluded meaningful comparisons across modalities. Regarding the minimum sample size, considering differences in treatment availability and the volume of published literature, the threshold for inclusion was set at ≥50 cases for CF-Photon RT, Linac-based SRS/SRT, and GKRS, and ≥10 cases for CF-PT.

### 2.2. Search Strategy and Study Selection

For selection of CF-PT articles, a PubMed search using the terms (proton beam therapy OR proton radiotherapy) AND meningioma identified 192 articles published between 2000 and 2024. Of these, 27 articles describing PT for meningioma were selected based on their titles or abstracts. After reviewing the full texts, studies with ≥10 cases that reported LC outcomes for WHO Grade 1 meningioma were selected. Articles were then excluded if they included combined photon radiotherapy, showed substantial patient-selection bias, or contained overlapping datasets, resulting in the final selection of six eligible studies [12,13,14,15,16,17].

**Figure 1:**
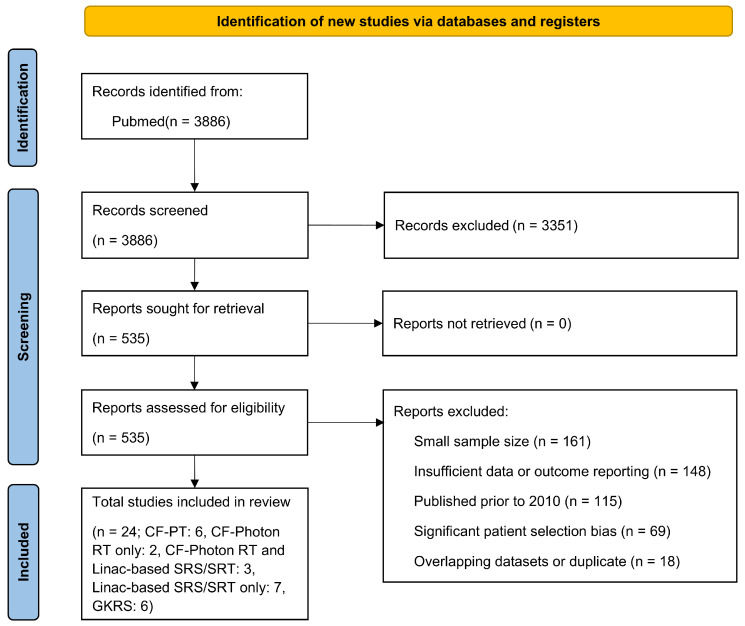
Flowcharts of study selection. CF-PT: Conventionally fractionated proton therapy; CF-Photon RT: Conventionally fractionated photon radiotherapy; Linac-based SRS/SRT: Linac-based stereotactic radiosurgery/radiotherapy; GKRS: Gamma Knife radiosurgery.

For CF-Photon RT, Linac-based SRS/SRT, and GKRS, a PubMed search using the terms (radiotherapy OR radiation therapy) AND meningioma identified 3694 articles published between 2000 and 2024. Of these, 508 articles describing radiotherapy for meningioma were selected based on their titles or abstracts. Because all eligible CF-PT studies were published from 2010 onwards, inclusion of studies evaluating other modalities was restricted to publications from 2010 onwards to improve consistency with contemporary treatment technologies. Among these, 228 prospective or retrospective studies including ≥50 patients were identified. Studies were further selected if they reported dose fractionation, treatment modality, and LC outcomes for WHO Grade 1 meningioma. After excluding studies involving particle therapy, patient-selection bias, or overlapping datasets, 18 studies remained (CF-Photon RT only: 2, CF-Photon RT and Linac-based SRS/SRT: 3, Linac-based SRS/SRT only: 7, GKRS: 6) [18,19,20,21,22,23,24,25,26,27,28,29,30,31,32,33,34,35]. The Linac-based SRS/SRT group included both conventional Linac systems and CyberKnife. The study selection process is summarized in Figure 1.

### 2.3. Data Extraction

Data extracted from the selected articles included author, publication year, follow-up period, mortality, local recurrence, 1- to 5-year LC rates, treatment modality, total dose, number of fractions, age, sex, and gross tumor volume (GTV). Additionally, 1- to 5-year overall survival (OS) data were collected for the CF-PT cohort. When 1- to 5-year LC or OS rates were not explicitly reported, values were estimated from published Kaplan–Meier curves or graphical data.

### 2.4. Statistical Analysis

Random-effects meta-analyses of 1- to 5-year LC rates were performed for each treatment modality, and forest plots were generated. For studies with missing precision estimates, values were statistically imputed using the number of patients, the risk set size at each time point, and the mean dropout rate [36]. Heterogeneity among studies was assessed using the I-square (I^2^) statistic. To compare outcomes among treatment modalities, random-effects meta-regression analyses were performed using treatment modality as an explanatory variable. Additional meta-regression analyses were conducted using age, sex ratio, and GTV as covariates. All analyses were performed using R ver. 4.6.0 (R Core Team, Vienna, Austria) and the meta package ver. 8.5-0 [37].

## 3. Results

Patient characteristics according to treatment modality are summarized in Table 1. The median ages of patients treated with CF-PT, CF-Photon RT, Linac-based SRS/SRT, and GKRS were 53, 58, 58, and 55 years, respectively. The proportion of male patients was also comparable among the four groups (25.0%, 23.0%, 25.7%, and 22.0%, respectively). In contrast, median GTV differed across modalities. The median GTV was 15.4 mL for CF-PT and 18.6 mL for CF-Photon RT, compared with 5.0 mL and 4.8 mL for Linac-based SRS/SRT and GKRS groups, respectively. Thus, conventionally fractionated radiotherapy tended to be used for larger tumors than stereotactic techniques. The median follow-up periods for the CF-PT, CF-Photon RT, Linac-based SRS/SRT, and GKRS were 69.5, 81.0, 64.7, and 72.0 months, respectively. The corresponding median prescription doses were 54.0, 53.4, 18.5, and 14.0 Gy.

**Figure 2:**
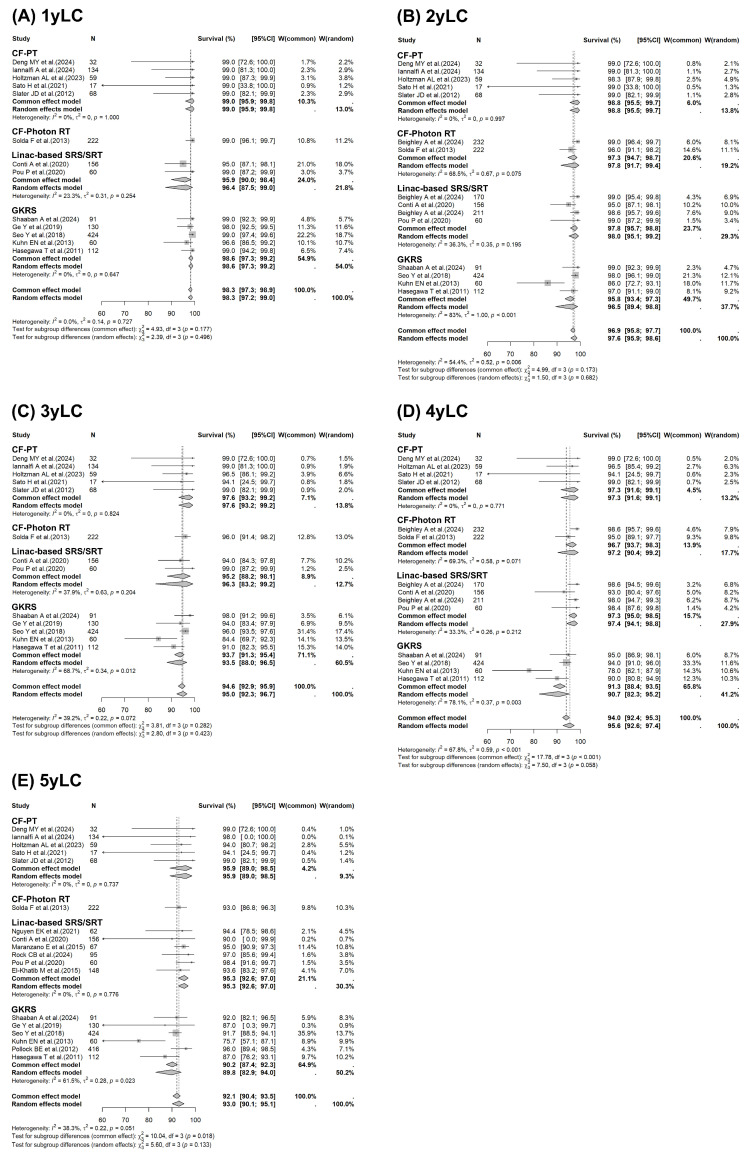
Forest plots of (**A**) 1-year, (**B**) 2-year, (**C**) 3-year, (**D**) 4-year, and (**E**) 5-year LC rates for CF-PT, CF-Photon RT, and Linac-based SRS/SRT, GKRS. LC: local control; CF-PT: Conventionally fractionated proton therapy; CF-Photon RT: Conventionally fractionated photon radiotherapy; Linac-based SRS/SRT: Linac-based stereotactic radiosurgery/radiotherapy; GKRS: Gamma Knife radiosurgery; CI: confidence interval [12,13,14,15,16,17,18,19,20,21,22,23,24,25,26,27,28,29,30,31,32,33,34,35].

Forest plots for 1- to 5-year LC rates for each modality are shown in Figure 2. Despite the differences in tumor volume, excellent LC was achieved across all treatment modalities. The pooled LC rates (95% confidence interval [CI]) for CF-PT, CF-Photon RT, Linac-based SRS/SRT, and GKRS were: 1-year LC: 99.0% (95.9–99.8%) vs. 99.0% (96.1–99.7%) vs. 96.4% (87.5–99.0%) vs. 98.6% (97.3–99.2%); 2-year LC: 98.8% (95.5–99.7%) vs. 97.8% (91.7–99.4%) vs. 98.0% (95.1–99.2%) vs. 96.5% (89.4–98.8%); 3-year LC: 97.6% (93.2–99.2%) vs. 96.0% (91.4–98.2%) vs. 96.3% (83.2–99.2%) vs. 93.5% (88.0–96.5%); 4-year LC: 97.3% (91.6–99.1%) vs. 97.2% (90.4–99.2%) vs. 97.4% (94.1–98.8%) vs. 90.7% (82.3–95.2%); and 5-year LC: 95.9% (89.0–98.5%) vs. 93.0% (86.8–96.3%) vs. 95.3% (92.6–97.0%) vs. 89.8% (82.9–94.0%).

Regarding tumor response, the reported tumor shrinkage rates were 39.3% (14.5–59%) for the Linac-based SRS/SRT and 34.8% (28.0–66.1%) for the GKRS, while no such data were available for the CF-PT or CF-Photon RT groups. Although the number of studies reporting clinical outcomes was limited, an improvement in pre-existing symptoms or quality of life (QOL) was observed in 29.4–37.5% for CF-PT, 27–37.5% for CF-Photon RT, 21.4–62% for Linac-based SRS/SRT, and 2.0–30.8% for GKRS.

Multivariable meta-regression analysis was performed using treatment modality, sex, age, and GTV as covariates. No significant association was observed between treatment modality and LC at any time point between 1 and 5 years. There was also no significant association between LC and GTV despite the larger tumor volumes in the conventionally fractionated radiotherapy cohorts. There was a significant association for mean patient age at 2 years (*p* = 0.0241) and 4 years (*p* = 0.0348), with an older age associated with slightly higher LC rates. No significant association was found between LC and sex at any of the evaluated time points (Table 2).

**Figure 3:**
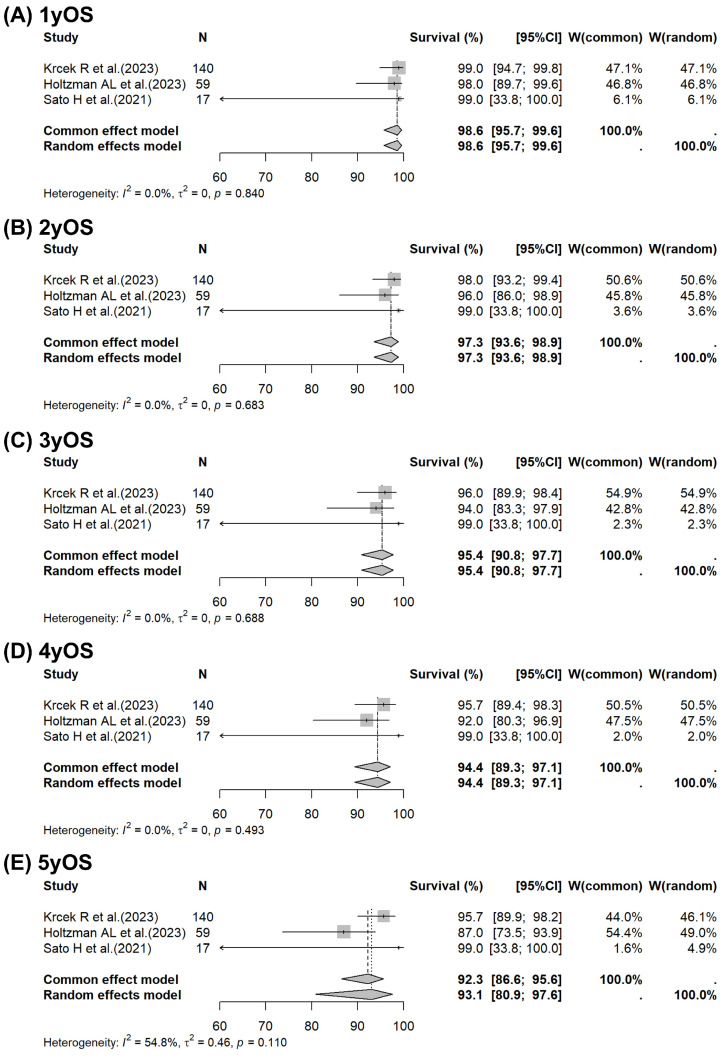
Forest plots of (**A**) 1-year, (**B**) 2-year, (**C**) 3-year, (**D**) 4-year, and (**E**) 5-year OS rates for CF-PT. OS: overall survival; CF-PT: Conventionally fractionated proton therapy; CI: confidence interval [13,14,15].

OS was additionally evaluated in the CF-PT cohort. The pooled OS rates (95% CI) were 98.6% (95.7–99.6%) at 1 year, 97.3% (93.6–98.9%) at 2 years, 95.4% (90.8–97.7%) at 3 years, 94.4% (89.3–97.1%) at 4 years, and 93.1% (80.9–97.6%) at 5 years. Forest plots for the 1- to 5-year OS rates after CF-PT are shown in Figure 3. Directly comparing OS with other modalities was beyond the scope of this analysis due to data constraints.

Treatment-related toxicities associated with each modality are summarized in Supplementary Table S1. Although several studies in the CF-PT group included patients with WHO grade 2 or 3 meningiomas in their toxicity reports, the data included in the present meta-analysis were derived from patients with WHO grade 1 meningiomas. Overall, the incidence of severe adverse events was low. Late toxicities reported in multiple studies included peritumoral edema, cranial neuropathy, radionecrosis, and arterial occlusion. One large GKRS study reported three treatment-related deaths (grade 5); however, treatment-related mortality was rare across the included studies.

## 4. Discussion

To our knowledge, this is the first study to compare LC outcomes among CF-PT, CF-Photon RT, Linac-based SRS/SRT, and GKRS for WHO Grade 1 meningioma. LC was excellent across all modalities, with 5-year LC rates exceeding 89%. Given the expected favorable long-term survival for most patients with WHO Grade 1 meningioma, these findings suggest that treatment selection should be guided by considerations of long-term survivorship, including treatment-related toxicity, in addition to tumor control. In the CF-PT cohort, most acute adverse events were mild (Grade 2 or less), and none necessitated treatment discontinuation. Regarding late adverse events, Grade 3 or higher radiation necrosis occurred at a low rate (0–2.4%), which was considered an acceptable rate. CF-PT achieved LC comparable to that of contemporary photon-based approaches while maintaining excellent OS, supporting its role as an effective treatment option for patients with WHO Grade 1 meningioma requiring radiotherapy.

In meta-regression analysis, older age was associated with improved LC at 2 years (*p* = 0.0241) and 4 years (*p* = 0.0348). However, this association was not consistent across all evaluated time points, and should therefore be interpreted cautiously. Furthermore, it should be noted that the annual LC rates in this analysis are results of independent pooled analyses at each time point, rather than longitudinal changes tracking a single cohort. Given the overall high control rates for WHO Grade 1 meningioma, slight variations in the reported values of individual papers may have influenced the estimates at each time point. Previous studies have reported conflicting findings for the influence of age on LC [22,25,38]. One possible explanation is that meningiomas in younger patients may exhibit greater proliferative potential and a higher likelihood of radiographic progression [4], whereas tumors in elderly patients often show slower growth kinetics. Nevertheless, given the indolent nature of WHO Grade 1 meningioma and the limited follow-up available in many studies, additional long-term investigations are needed before definitive conclusions on age-related differences in tumor control can be drawn.

A notable finding of this study was the difference in tumor volume among treatment modalities. The median GTV for CF-PT (15.4 mL) and CF-Photon RT (18.6 mL) was considerably larger than that for Linac-based SRS/SRT (5.0 mL) and GKRS (4.8 mL), a difference that became even more apparent when maximum tumor volumes were compared (296.0–754.0 vs. 51.8–62.7 mL). This likely reflects routine clinical practice, in which conventionally fractionated radiotherapy is selected for larger tumors or lesions adjacent to critical structures, whereas stereotactic techniques are generally reserved for smaller lesions. Beighley et al. reported that a tumor volume > 5 mL was associated with reduced LC [22], but GTV was not significantly associated with LC in our adjusted analysis. While the impact of tumor volume may have been underestimated due to limited statistical power or collinearity between modality and tumor size, the clinical implication remains important: CF-PT and CF-Photon RT achieved LC rates comparable to those of stereotactic approaches despite treating larger tumors. These findings suggest that modern high-precision radiotherapy can compensate for the challenges associated with larger tumor volumes and maintain durable tumor control across a broad range of clinical scenarios.

Treatment-related toxicity is an important consideration for patients with WHO Grade 1 meningioma. Because these tumors are benign and most patients have prolonged survival, minimizing late adverse effects may be as important as achieving durable tumor control. Tumor volume is associated with a risk of treatment-related toxicity [38], and treatment of large or anatomically complex lesions often necessitates irradiation of substantial volumes of normal brain tissue. Consequently, concerns extend beyond acute adverse events to include long-term complications such as cerebrovascular disease and neurocognitive decline. In this context, PT offers several potential advantages. Owing to the physical properties of the Bragg peak, PT can reduce unnecessary radiation exposure to surrounding normal tissues. Florijn et al. compared treatment plans for skull base meningiomas of ≥3 cm and found that intensity-modulated proton therapy (IMPT) significantly reduced the mean dose to normal brain tissue and the hippocampus compared with non-coplanar volumetric modulated arc therapy (VMAT) and intensity-modulated radiotherapy (IMRT) [39]. In clinical practice, a study comparing brain volume changes after photon and proton therapy for brain tumors reported that the volume of atrophy was greater with photon therapy [40]. Furthermore, Sherman et al. prospectively followed 20 patients with low-grade glioma treated with PT over five years and reported that cognitive function remained stable overall, with no significant decline [41]. A clinical trial is also currently underway to compare toxicity outcomes between PT and IMRT for glioblastoma [42]. Regarding meningioma, Krcek et al. also reported that QOL is maintained over the long term after proton therapy. Collectively, these studies suggest that reducing radiation exposure to normal brain tissue may contribute to the long-term maintenance of neurocognitive function and QOL. These dosimetric advantages may be particularly important in patients requiring irradiation of large target volumes or lesions adjacent to a critical organ at risk.

Several limitations of this study should be acknowledged. First, all included studies were retrospective single-center studies, and selection bias could not be completely excluded. Furthermore, there was variability in endpoints and the reporting of adverse-event among studies, and insufficient data were available to permit direct comparisons of OS, progression-free survival and adverse-event profiles among modalities. Although CF-PT demonstrated acceptable toxicity and gave LC rates comparable to other approaches despite treating larger tumors, future comparative studies should evaluate clinically relevant endpoints such as radiation-induced edema, radiation necrosis, cerebrovascular events, neurocognitive decline, and longitudinal QOL outcomes. Such endpoints may ultimately prove more important than tumor control alone in patients with benign tumors and prolonged life expectancy.

Second, follow-up durations were limited. Assessment of late effects such as cerebrovascular disease and neurocognitive decline requires observation over many years. Moreover, WHO Grade 1 meningiomas may recur 10–20 years or more after treatment. Thus, 5-year LC rates should be interpreted as intermediate-term outcomes rather than definitive measures of cure. In addition, this study focused on LC as the primary endpoint and did not evaluate tumor regression. Chung et al. reported higher radiographic shrinkage rates after stereotactic radiosurgery compared with fractionated radiotherapy [38]. Future studies should assess both tumor control and volumetric response and associated clinical improvements, including recovery of neurological symptoms.

Third, there was heterogeneity among the included studies. The analyzed cohorts encompassed definitive radiotherapy for unresected tumors, adjuvant radiotherapy after subtotal resection, and salvage radiotherapy for recurrent disease. Studies with extreme imbalances in treatment indication were excluded to improve comparability, but detailed subgroup analyses according to treatment background were not feasible. Additionally, information regarding surgical status, recurrent disease, tumor location, pathological diagnosis and molecular classification was inconsistently reported across studies, precluding further quantitative analyses of these factors. Recent advances in molecular biology have shifted the management of meningiomas from conventional histological grading toward an integrated classification incorporating genetic alterations and methylation profiles. These molecular classifications are expected to improve prognostic stratification, guide the selection of molecularly targeted therapies, and identify patients who are more likely to benefit from radiotherapy. However, clinical evidence supporting these personalized treatment strategies remains limited. Future studies should evaluate the long-term toxicities and patient outcomes associated with advanced radiotherapy techniques, including proton beam therapy, while accumulating clinical data based on molecular classification. Treatment strategies integrating radiotherapy with molecular classification will be needed to optimize long-term survivorship in patients with meningioma.

## 5. Conclusions

Major radiotherapy modalities for WHO Grade 1 meningioma, including CF-PT, achieved excellent and comparable 5-year LC despite differences in tumor volume and treatment selection patterns. Notably, conventionally fractionated radiotherapy maintained favorable tumor control despite being applied to larger tumors than stereotactic approaches. Given the prolonged survival expected in this patient population, future research should focus on long-term tumor control and treatment-related toxicity, recovery of neurological symptoms and QOL to define optimal treatment strategies.

## Figures and Tables

**Table 1:** Backgrounds of patients.

Factor	CF-PT	CF-Photon RT	Linac-Based SRS/SRT	GKRS
Total patients	450	1544	1446	1233
Age				
Median (years)	53	58	58	55
Range	8–85	10–86	10–87	8–90
Male				
Median (%)	25.0	23.0	25.7	22.0
Range	22.0–35.3	18.0–24.1	15.0–72.0	17.7–28.0
Gross Tumor Volume				
Median (mL)	15.4	18.6	5.0	4.8
Range	0–296.0	0.4–754.0	0.1–51.8	0–62.7
Median follow-up period				
Median (months)	69.5	81.0	64.7	72.0
Range	8.4–204.0	1.0–349	1.0–259.2	4.0–234.0
Total dose				
Median (Gy)	54.0	53.4	18.5	14.0
Range	45.0–66.6	32.4–66.2	5.0–61.0	7.0–20.4
Number of Fractions				
Median (fractions)	28	30	1	1
Range	25–37	24–42	1–15	1–1

CF-PT: Conventionally fractionated proton therapy; CF-Photon RT: Conventionally fractionated photon radiotherapy; Linac-based SRS/SRT: Linac-based stereotactic radiosurgery/radiotherapy; GKRS: Gamma Knife radiosurgery.

**Table 2:** Meta-regressions of potential predictive factors for 1- to 5-year local control.

	Estimate	SE	Z Value	*p*-Value	CI.LB	CI.UB
1-year LC						
CF-PT	6.5912	10.0632	0.6550	0.5125	−13.1324	26.3148
CF-Photon RT	0.0257	1.2580	0.0204	0.9837	−2.4399	2.4912
Linac-based SRS/SRT	−1.1869	1.8889	−0.6283	0.5298	−4.8892	2.5154
GKRS	−0.0563	1.5255	−0.0369	0.9706	−3.0462	2.9336
Male Ratio	−0.0129	0.1263	−0.1019	0.9189	−0.2605	0.2347
Age	−0.0341	0.2161	−0.1578	0.8746	−0.4577	0.3895
GTV	0.0044	0.0918	0.0476	0.9621	−0.1755	0.1842
2-year LC						
CF-PT	0.0879	3.1297	0.0281	0.9776	−6.0462	6.2220
CF-Photon RT	−1.1035	0.9354	−1.1797	0.2381	−2.9367	0.7298
Linac-based SRS/SRT	−1.7053	1.0694	−1.5947	0.1108	−3.8012	0.3906
GKRS	−0.6479	1.0264	−0.6313	0.5279	−2.6597	1.3638
Male Ratio	−0.1513	0.0958	−1.5791	0.1143	−0.3392	0.0365
Age	0.1362	0.0604	2.2560	0.0241	0.0179	0.2544
GTV	0.0563	0.0838	0.6722	0.5015	−0.1079	0.2205
3-year LC						
CF-PT	13.9857	8.5266	1.6402	0.1010	−2.7262	30.6975
CF-Photon RT	−0.4026	0.7554	−0.5330	0.5941	−1.8832	1.0780
Linac-based SRS/SRT	0.4143	1.2107	0.3422	0.7322	−1.9585	2.7872
GKRS	−0.1958	0.8437	−0.2321	0.8165	−1.8494	1.4578
Male Ratio	−0.0279	0.0929	−0.3004	0.7639	−0.2100	0.1542
Age	−0.1848	0.1845	−1.0018	0.3165	−0.5464	0.1768
GTV	−0.0110	0.0636	−0.1728	0.8628	−0.1356	0.1136
4-year LC						
CF-PT	0.2190	2.6553	0.0825	0.9343	−4.9854	5.4233
CF-Photon RT	−0.4364	0.7541	−0.5788	0.5627	−1.9144	1.0415
Linac-based SRS/SRT	−1.1003	0.8864	−1.2413	0.2145	−2.8376	0.6370
GKRS	−1.0496	0.7824	−1.3415	0.1797	−2.5831	0.4839
Male Ratio	−0.1305	0.0697	−1.8718	0.0612	−0.2671	0.0061
Age	0.1141	0.0540	2.1111	0.0348	0.0082	0.2200
GTV	0.0268	0.0590	0.4540	0.6499	−0.0889	0.1425
5-year LC						
CF-PT	3.5586	6.1631	0.5774	0.5637	−8.5208	15.6380
CF-Photon RT	−0.3873	0.7835	−0.4943	0.6211	−1.9230	1.1484
Linac-based SRS/SRT	0.3160	0.9271	0.3409	0.7332	−1.5010	2.1331
GKRS	−0.4129	0.8370	−0.4933	0.6218	−2.0534	1.2276
Male Ratio	−0.0560	0.0610	−0.9173	0.3590	−0.1756	0.0636
Age	0.0082	0.1190	0.0687	0.9452	−0.2252	0.2415
GTV	0.0262	0.0535	0.4891	0.6248	−0.0787	0.1310

LC: Local control; CF-PT: Conventionally fractionated proton therapy; CF-Photon RT: Conventionally fractionated photon radiotherapy; Linac-based SRS/SRT: Linac-based stereotactic radiosurgery/radiotherapy; GKRS: Gamma Knife radiosurgery; GTV: gross tumor volume; SE: standard error; CI.LB: confidence interval lower bound; CI.UB: confidence interval upper bound.

## Data Availability

All data generated or analyzed during this study are included in this published article and its Supplementary Materials.

## Supplementary Materials

The following supporting information can be downloaded at: https://www.mdpi.com/article/10.3390/cancers18152506/s1, Table S1. Summary of Acute and Late Toxicities Following Radiotherapy for Meningioma.

## Abbreviations

The following abbreviations are used in this manuscript:

WHO	World Health Organization
PT	Proton therapy
CF-PT	Conventionally fractionated proton therapy
CF-RT	Conventionally fractionated photon radiotherapy
Linac-based SRS/SRT	Linac-based stereotactic radiosurgery/radiotherapy
GKRS	Gamma Knife radiosurgery
GTV	Gross tumor volume
LC	Local control
OS	Overall survival
