# Supplementary material for: Radiotherapy Strategies for WHO Grade 1 Meningioma: A Systematic Review and Meta-Analysis Comparing Proton Therapy, Conventional Radiotherapy, and Radiosurgery
Source: Cancers (Basel). 2026 Aug 5;18(15):2506. doi: 10.3390/cancers18152506 (PMC13465174; doi:10.3390/cancers18152506)
Supplement: Supplementary file 1 [file cancers-18-02506-s001.zip › cancers-4445742-supplementary.pdf]

Supplementary Table S1. Summary of Acute and Late Toxicities Following Radiotherapy for Meningioma

Modality	Author, Year	n	Acute Toxicity	Late Toxicity
CF-PT	Deng, 2024	32	Grade 1–2: Fatigue 7 (21.9%), Cephalgia 7 (21.9%), Vertigo 3 (9.4%), Lacrimal dysfunction 3 (9.4%), Nausea 1 (3.1%).<br>No Grade ≥3.	Vertigo 3 (9.4%), Fatigue 4 (12.5%), Cephalgia 6 (18.8%), Lacrimal dysfunction 2 (6.3%), Decreased visual acuity 4 (12.5%), Visual field loss 4 (12.5%), RION 3 (9.4%).<br>No Grade ≥4.
CF-PT	*Iannalfi, 2024	167	Including alopecia, radiation dermatitis, oral mucositis, soft tissue edema.	Grade 2: Optic nerve toxicity 2 (1.2%), Radionecrosis 7 (4.2%), Endocrine toxicity 3 (1.8%), Cranial nerve toxicities.<br>Grade 3: Optic nerve toxicity 2 (1.2%), Radionecrosis 4 (2.4%), Endocrine toxicity (NA).
CF-PT	*Krcek, 2023	200	Alopecia, Dermatitis, Fatigue, Headache, Nausea.<br>No Grade ≥3.	Grade ≥ 3: Visual toxicity 10 (5.0%), Cataract 4 (2.0%), Brain necrosis 3 (1.5%), Ear/labyrinth disorder 2 (1.0%), Stroke 2 (1.0%), Brain edema 1 (0.5%), Pain 1 (0.5%), Pituitary dysfunction 1 (0.5%).
CF-PT	Holtzman, 2023	59	NA	Grade 3: Vision loss 1 (1.7%), Secondary neoplasm 1 (1.7%).<br>No Grade ≥4.
CF-PT	Sato, 2021	17	Grade 1–2: Radiation dermatitis 7 (41.2%), Alopecia 2 (11.8%), Vomiting 2 (11.8%), Middle ear inflammation 2 (11.8%), Cephalgia 1 (5.9%), Mucositis oral 1 (5.9%), Gastritis 1 (5.9%).<br>No Grade ≥3.	0 (0%)
CF-PT	*Slater, 2012	72	NA	Neurologic (visual) symptoms 3 (4.2%), Panhypopituitarism 3 (4.2%), Brain edema 2 (2.8%), Transient diplopia 1 (1.4%).
CF-Photon RT	Beighley, 2024	232	NA	Peritumoral edema 28 (12.1%), Cranial neuropathies 5 (2.2%).
CF-Photon RT	Conti, 2019	136	Grade ≥3: Brain edema 1 (0.7%).	Grade 1–2: Cranial neuropathy 12 (8.8%), Seizures 1 (0.7%).<br>Grade ≥3: Vascular stenosis 2 (1.5%).

CF-Photon RT	Combs, 2018	808	No Grade ≥3.	Cranial nerve impairment.
CF-Photon RT	Solda, 2013	222	Localized alopecia 222 (100.0%), Headache 8 (3.6%), Visual deterioration 3 (1.4%).	Worsening vision 8 (3.6%), Radiation retinopathy 2 (0.9%), Cognitive impairment 2 (0.9%), Cerebrovascular accident 2 (0.9%), Trigeminal neuralgia 1 (0.5%), Retinal artery occlusion 1 (0.5%), TIA 1 (0.5%).
CF-Photon RT	Tanzler, 2011	146	NA	Ophthalmologic complications 4 (2.7%), Radionecrosis 2 (1.4%), Peritumoral edema 2 (1.4%), Hydrocephalus 1 (0.7%), Osteomyelitis of external ear canal 1 (0.7%).
Linac-based SRS/SRT	Beighley (SRT), 2024	170	NA	Peritumoral edema 36 (21.2%), Cranial neuropathies 1 (0.6%).
Linac-based SRS/SRT	Nguyen (SRT), 2021	62	NA	Radionecrosis 3 (4.8%).
Linac-based SRS/SRT	Conti (SRT), 2020	156	NA	Cranial neuropathy 9 (5.8%), Peritumoral edema 1 (0.6%), Carotid artery occlusion 1 (0.6%).
Linac-based SRS/SRT	Huang (SRT), 2020	81	NA	NA
Linac-based SRS/SRT	Conti (SRT), 2019	205	No Grade ≥3.	Grade 1–2: Cranial neuropathy 16 (7.8%).<br>Grade ≥3: Carotid artery occlusion 1 (0.5%), Transient facial nerve deficit 1 (0.5%).
Linac-based SRS/SRT	Taranzano (SRT), 2015	67	No Grade ≥3.	No Grade ≥3.
Linac-based SRS/SRT	Beighley (SRS), 2024	211	NA	Peritumoral edema 14 (6.6%), Cranial neuropathies 2 (0.9%).
Linac-based SRS/SRT	Rock (SRS), 2024	95	NA	NA

Linac-based SRS/SRT	Huang (SRS), 2020	72	NA	NA
Linac-based SRS/SRT	Pou (SRS), 2020	60	NA	Motor disability 4 (6.7%), Seizures 2 (3.3%), Peritumoral edema 5 (8.3%), Visual disability 1 (1.7%).
Linac-based SRS/SRT	Combs (SRS), 2018	119	No Grade ≥3.	Cranial nerve impairment.
Linac-based SRS/SRT	El-Khatib (SRS), 2015	148	NA	Seizures 5 (3.4%), Radiation-induced edema 3 (2.0%), Trigeminal neuralgia 3 (2.0%), Cephalgia 2 (1.4%).
GKRS	Shaaban, 2024	91	NA	Cranial nerve deficit 6 (6.6%).
GKRS	Ge, 2019	130	Nausea/Vomiting 2 (1.5%), Visual acuity deterioration 1 (0.8%), Facial paralysis 1 (0.8%), Facial numbness 1 (0.8%).	Peritumoral edema 6 (4.6%), Cranial neuropathies 3 (2.3%).
GKRS	Seo, 2018	424	NA	Grade 1–2: Headache 17 (4.0%), Dizziness/Nausea 7 (1.7%), Visual disturbance 4 (0.9%), Ptosis 1 (0.2%), Diplopia 3 (0.7%), Facial hypesthesia 4 (0.9%), Facial pain 7 (1.7%), Facial palsy 1 (0.2%), Seizure 3 (0.7%), Motor weakness 3 (0.7%), Other 1 (0.2%).<br>Grade 3: Facial pain 2 (0.5%), Visual disturbance 1 (0.2%), Seizure 1 (0.2%), Motor weakness 1 (0.2%), Other 2 (0.5%).<br>NA Grade: Peritumoral edema 65 (15.3%).<br>Grade 5: 1 (0.2%).
GKRS	Kuhn, 2013	60	NA	NA

GKRS	Pollock, 2012	416	NA	Hemiparesis 10 (2.4%), Seizures 10 (2.4%), Facial pain 9 (2.2%), Facial numbness 7 (1.7%), Diplopia 4 (1.0%), Hearing loss 2 (0.5%), Headache 2 (0.5%), Facial weakness 1 (0.2%), Hemifacial spasm 1 (0.2%), Symptomatic cyst 1 (0.2%), Ischemic stroke/ICA occlusion 1 (0.2%), Pontine cavernous malformation 1 (0.2%).<br>Grade 5: 3 (0.7%).
GKRS	Hasegawa, 2011	112	NA	Peritumoral edema 29 (25.9%), Secondary neoplasm 1 (0.9%).

CF-PT: Conventionally fractionated proton therapy; CF-Photon RT: Conventionally fractionated photon radiotherapy; Linac-based SRS/SRT: Linac-based stereotactic radiosurgery/radiotherapy; GKRS: Gamma Knife radiosurgery; RION: Radiation-Induced Optic Neuropathy; TIA: Transient Ischemic Attack; ICA: Internal Carotid Artery; NA: Not Available.

* Studies included cases with WHO Grade 2 and 3; only data for WHO Grade 1 were extracted for this analysis.
